# Experience with second allogeneic hematopoietic stem cell transplantation in Chilean patients: A single-center study

**DOI:** 10.1016/j.htct.2025.103980

**Published:** 2025-10-04

**Authors:** Yorman Flores, Javier Díaz, Patricio Rojas, José Salinas, Catherine Gutiérrez, Marcela Vidal, Verónica Jara, Elizabeth Rivera, María José García, Vicente Sandoval, Felipe Palacios, Maximiliano Vergara, Mauricio Ocqueteau, Mauricio Sarmiento

**Affiliations:** Adult Hematopoietic Transplant Program, Red de Salud UC Christus, Pontificia Universidad Católica de Chile, Chile

**Keywords:** Leukemia, Aplastic anemia, Myelodysplastic syndromes, Allogeneic stem cell transplantation

## Abstract

**Introduction:**

Allogeneic hematopoietic stem cell transplantation is potentially a curative treatment for several hematological diseases. However, post-transplant relapse remains a significant challenge. For patients who achieve a second complete remission, a second allogeneic transplantation may be a promising therapeutic option. The aim of this study was to analyze clinical outcomes including graft-versus-host disease, non-relapse mortality, and relapse rates, as well as graft sources in patients who underwent a second allogeneic transplantation in a university-based transplant program.

**Patients and Methods:**

A retrospective analysis of 21 adult patients who underwent a second allogeneic transplantation between 2001 and 2023 was performed. Data on demographics, underlying disease, graft source, conditioning, graft-versus-host disease, relapse, and survival were collected. Survival estimates were calculated using the Kaplan–Meier method.

**Results:**

The graft source was bone marrow in 60 % and peripheral blood in 40 % of cases. Grade III–IV acute graft-versus-host disease occurred in 5 % and extensive chronic graft-versus-host disease in 17 %. The non-relapse mortality was 69.2 %, and disease relapse occurred in 23.1 %. The one-year progression-free survival was 26.5 %, and overall survival was 42.3 %. Compared to those transplanted before 2010, patients who underwent transplantation after 2010 showed improved two-year PFS and OS, reaching 55 % and 45.4 %, respectively.

**Conclusion:**

A second allogeneic transplantation may offer a survival benefit in selected patients with relapsed hematologic malignancies or bone marrow failure syndromes. Despite high non-relapse mortality, outcomes have improved in recent years with better salvage strategies.

## Introduction

Allogeneic hematopoietic stem cell transplantation (HSCT) is administered with curative intent in multiple hematologic disorders. However, a substantial proportion of patients relapse following this treatment. A second allogeneic transplantation (ALO2) is an option for a subgroup of patients who achieve remission after relapse, as demonstrated by leading transplant centers, predominantly in developed countries [1]. In Chile, the National Public Health Transplantation Program does not offer an ALO2 to patients who experience relapse after HSCT. Nevertheless, university and private centers provide ALO2 to fit patients who achieve a second remission.

Patients who relapse after an initial HSCT have a dismal prognosis and poor long-term survival [2,3]. Recent developments in salvage therapies have made it possible for selected patients to achieve remission and proceed to ALO2. In this context, the objective of this study was to analyze the outcomes of patients undergoing ALO2 in a Chilean university hospital, focusing on survival, complications, and treatment feasibility.

## Methods

This was a retrospective, descriptive study conducted in patients aged 18 years or older who underwent a ALO2 between 2001 and 2023 at the Hematology Department of Red de Salud UC Christus, Pontificia Universidad Católica de Chile.

The primary outcomes were overall survival (OS) and progression-free survival (PFS), estimated using Kaplan–Meier methodology and the secondary outcomes were incidence of acute and chronic graft-versus-host disease (GvHD), non-relapse mortality (NRM), and relapse. GvHD was defined and graded per standard criteria.

Data on demographics, underlying hematologic disease, conditioning regimens, donor source, CD34^+^ cell dose, engraftment, complications, and cause of death were obtained from clinical records and the transplant program database.

The study protocol was approved by the institutional review board of the Pontificia Universidad Católica de Chile.

## Results

A total of 1203 patients underwent HSCT at the center between 2001 and 2023. Of these, 602 (51 %) received an allogeneic transplant. Of this group, 21 patients underwent a ALO2 due to relapse or graft failure. The median age was 33.4 years (Range: 18–59 years), and 42 % were female.

The graft source was bone marrow in 60 % and peripheral blood in 40 % of cases. Human leukocyte antigen (HLA)-identical family donors were used in 55 %, haploidentical in 20 %, and unrelated donors in 25 %. The characteristics of the patients are summarized in Table 1.

**Table 1:** Patient characteristics.

Characteristic	
Gender -%	
Male	57.9
Female	42.1
Median Age (years) – median (range)	30
Hematologic Disorder - n (%)	
Acute Myeloid Leukemia	8 (38)
Acute Lymphoblastic Leukemia	5 (23)
Chronic Myeloid Leukemia	2 (8)
Hodgkin Lymphoma	3 (13)
Non-Hodgkin Lymphoma	1 (5)
Severe Aplastic Anemia	3 (13)
Donor Type - n	
HLA-Identical Family	11
Haploidentical	4
Unrelated	5
Stem Cell Source -%	
Bone Marrow	60
Peripheral Blood	40

Conditioning regimens varied by period. Between 2001 and 2010, regimens included busulfan/cyclophosphamide, total body irradiation (TBI)/etoposide (Etoposide)/cyclophosphamide, and fludarabine/cyclophosphamide. From 2011 to 2023, regimens included fludarabine/cyclophosphamide/TBI, fludarabine/busulfan, fludarabine/treosulfan, and cyclophosphamide/ anti-thymocyte globulin (ATG).

GvHD prophylaxis used calcineurin inhibitors in all patients, methotrexate (Methotrexate) in 71 %, and post-transplant cyclophosphamide in 29 %. The mean CD34^+^ cell dose was 6.62 × 10⁶/kg bodyweight (95 % CI: 2.9–11.0). Median neutrophil and platelet engraftment times were 16 days (range: 8–35 days) and 17 days (range: 9–75 days), respectively.

Grade III–IV acute GvHD occurred in 5 %, and extensive chronic GvHD in 17 % of the cases. NRM was 69.2 %, while disease relapse accounted for 23.1 % of deaths. The median follow-up time was 13.5 months. Transplantation data are summarized in Table 2.

**Table 2:** Transplantation Characteristics.

**Characteristic**	
Conditioning Regimen (2001–2010) - n	
Busulfan, Cyclophosphamide	4
TBI 100 Gy, Etoposide Cyclophosphamide	3
Fludarabine, Cyclophosphamide	1
Conditioning Regimen (2011–2023) - n	
Flu-Cy-TBI (200–400 Gy)	4
Fludarabine, Busulfan	3
Flu-Treosulfan	2
Cyclophosphamide, ATG	3
GvHD Prophylaxis - n	
Calcineurin Inhibitors	21
Methotrexate	15
Post-Transplant Cyclophosphamide	6
CD34^+^ Cell Dose (× 10⁶/kg bodyweight) - median (range)	6.62 (2.9–11.6)
Granulocyte Engraftment (days) - median (range)	16 (8–35)
Platelet Engraftment (days) - median (range)	17 (9–75)
Acute GvHD Grade 3–4 -%	5
Extensive Chronic GvHD -%	17

Flu: fludarabine; ATG: anti-thymocyte globulin; Gy: grays, Cy: cyclophosphamide; TBI: total body irradiation; GvHD: graft-versus-host diseases.

**Figure 1:**
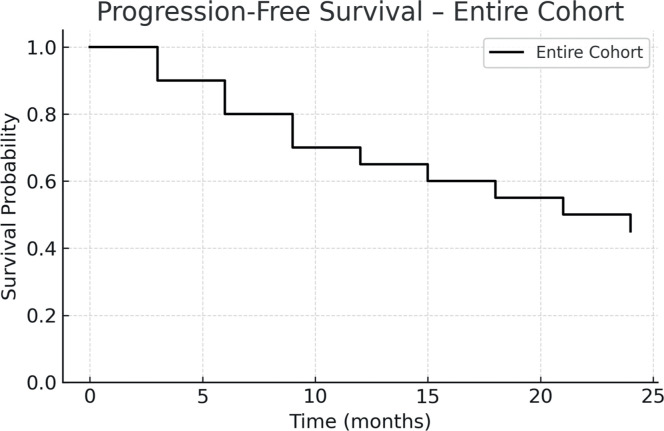
Progression-free survival in patients undergoing second allogeneic hematopoietic stem cell transplantation.

**Figure 2:**
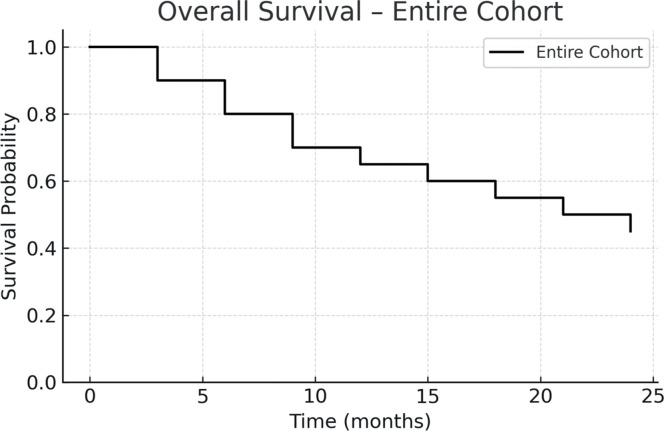
Overall Survival in patients undergoing second allogeneic hematopoietic stem cell transplantation.

**Figure 3:**
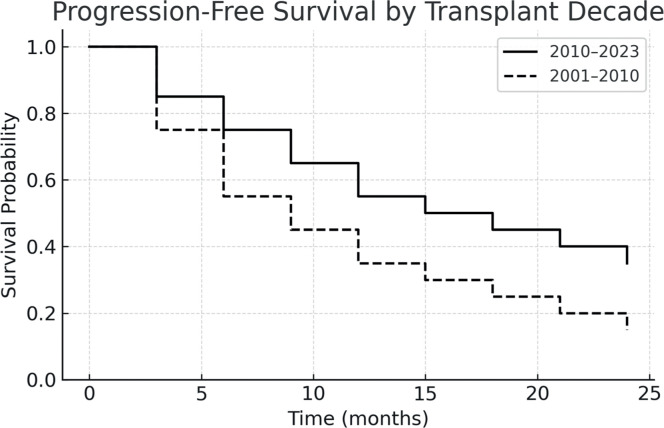
Progression-free survival in patients undergoing second allogeneic hematopoietic stem cell transplantation according to the transplant decade.

**Figure 4:**
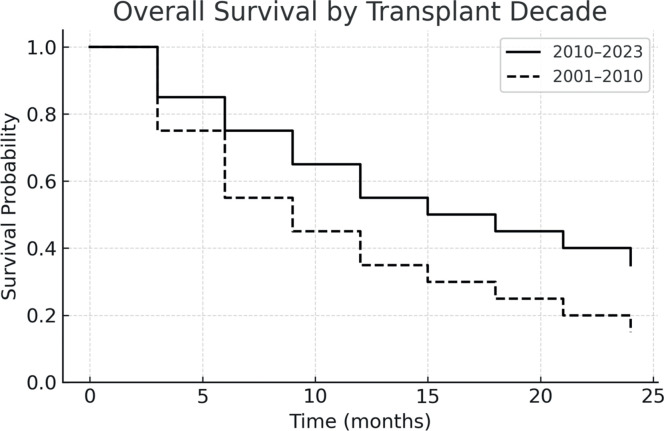
Overall Survival (OS) in patients undergoing second allogeneic hematopoietic stem cell transplantation according to the transplant decade.

The one-year PFS and OS were 26.5 % and 42.3 %, respectively. When stratified by transplant period, patients transplanted between 2010 and 2023 had improved outcomes: one-year PFS was 55 %, and OS was 45.4 %. In contrast, for patients treated between 2001 and 2010, both one-year PFS and OS were 12.5 %, with median survival times of 51 and 52 days, respectively. Figure 1, Figure 2, Figure 3, Figure 4 show the Kaplan Meier survival curves for PFS, OS, PFS by transplant decade and OS by transplant decade.

## Discussion

This retrospective study contributes to the limited data available from Latin America on the outcomes of an ALO2. While ALO2 is a well-established salvage option in developed countries, its role in resource-constrained settings is less defined.

There is currently no standard of care for patients who relapse after an initial allogeneic transplant. Treatment strategies are often individualized, depending on multiple variables including patient comorbidities, disease characteristics, access to salvage therapies, donor availability, and functional status at relapse [4,5]. Options include donor lymphocyte infusions, immunosuppression withdrawal, targeted agents, chimeric antigen receptor (CAR)-T therapies, and repeat transplantation. A ALO2 remains one of the few potentially curative strategies in selected patients, particularly those who achieve a second remission.

Multiple retrospective studies have shown that ALO2 can lead to long-term survival in a minority of patients. The Société Française de Greffe de Moelle (SFGM) [6] reported a two-year disease-free survival (DFS) and OS of 35 % and 41 %, respectively in 150 patients, 61 % of whom had acute myeloid leukemia (AML). Similarly, a Center for International Blood & Marrow Transplant (CIBMTR) Research report [7] found a three-year OS of 27 % in AML patients who underwent ALO2. In a European Society for Blood and Marrow Transplantation (EBMT) analysis [8], the NRM rates at two and five years were 24 % and 26 %, respectively. More recently, ALO2 has been used in combination with CAR-T cell therapy [9,10] to consolidate remission after relapse, with promising results, especially in relapsed/refractory B-cell malignancies. However, such strategies remain largely inaccessible in Latin American countries due to financial and logistical constraints.

The findings of this study demonstrate that survival after ALO2 is possible even in resource-limited settings. The one-year OS in the present cohort was 42.3 %, and PFS was 26.5 %, which aligns with international data. Notably, patients treated after 2010 had significantly better outcomes, suggesting improvements in patient selection, salvage therapy efficacy, and transplant protocols. However, non-relapse mortality was high (69.2 %) with infections and GvHD being major contributors. Another Latin-American experience [11] reported a 66 % mortality rate in 12 patients who underwent ALO2, highlighting similar challenges in the region. These findings emphasize the need for real-world data and local treatment strategies tailored to regional limitations.

Despite the inherent limitations of retrospective design and small sample size, this study provides valuable insights into the feasibility and outcomes of ALO2 in Latin America. Future prospective studies and international collaborations are needed to better define best practices and improve accessibility to potentially curative treatments in developing countries.

## Conflicts of interest

The author declares no conflicts of interest.
